# The Multi-temporal and Multi-dimensional Global Urban Centre Database to Delineate and Analyse World Cities

**DOI:** 10.1038/s41597-023-02691-1

**Published:** 2024-01-17

**Authors:** Michele Melchiorri, Sergio Freire, Marcello Schiavina, Aneta Florczyk, Christina Corbane, Luca Maffenini, Martino Pesaresi, Panagiotis Politis, Filip Szabo, Daniele Ehrlich, Pierpaolo Tommasi, Donato Airaghi, Luigi Zanchetta, Thomas Kemper

**Affiliations:** 1 European Commission, Joint Research Centre, Ispra, Italy; 2 Uni Systems, Luxembourg, Luxembourg; 3 Arhs Developments S.A., Belvaux, Luxembourg; 4 Fincons Group, Via Torri Bianche, 10, I-20871 Vimercate, (MB) Italy; 5 Engineering S.p.a, Piazzale dell’Agricoltura, 24, 00144 Roma, (RM) Italy

**Keywords:** Geography, Interdisciplinary studies, Sustainability

## Abstract

Monitoring sustainable urban development requires comparable geospatial information on cities across several thematic domains. Here we present the first global database combining such information with city extents. The Global Human Settlement Urban Centre Database (GHS-UCDB) is produced by geospatial data integration to characterise more than 10,000 urban centres worldwide. The database is multi-dimensional and multi-temporal, containing 28 variables across five domains and having multitemporal attributes for one or more epochs when the UC are delineated (1975-1990-2000-2015). Delineation of urban centres for the year 2015 is performed via a logic of grid cell population density, population size, and grid cell contiguity defined by the Degree of Urbanisation method. Each of the urban centres has 160 attributes, including a validation assessment. The novel aspects of this database concern the thematic richness and temporal depth of the variables (across geography, socio-economic, environmental, disaster risk reduction, and sustainable development domains) and the type of geo-information provided (location and extent), featuring an overall consistency that allows comparative analyses across locations and time.

## Background & Summary

In an increasingly urbanised world, cities are at the epicentres of the quest for global sustainability. the battle for sustainable development will be won or lost in cities^1^ given their significant environmental and socioeconomic footprint of cities.

This recent awareness drives the demand for data about the most populated places on Earth. For example, despite being common knowledge that the majority of world population lives in cities, there is no harmonised data for these places that adopts a harmonised delineation for the areas of interest. The pivotal role of cities is demonstrated by the existence of a specific Sustainable Development Goal on cities and human settlements (SDG 11), which also has many interlinkages with the other SDG^2^.The complexity of urban systems requires multi-thematic and spatially-explicit data for analyses and policy formulation.

Despite this importance, the availability of multi-thematic data on cities having global and multi-temporal coverage remained scarce, incomplete and fragmented due to limitations in thematic information (variables), and unstructured for lack of a harmonized definition of the areas of interest (i.e. cities). This ultimately undermined comparative, analytical purposes (see Table 1). Reviews of city definitions in use worldwide all conclude that quantitative analyses for cross-country comparisons are difficult and sometimes erroneous due to the diversity of concepts^3,4^ still affecting the currently available datasets.

**Table 1:** Relationship between observed data gaps, data features, design principles and solutions adopted for the production of GHS-UCDB.

Data gaps	Features	Principles	GHS-UCDB solutions
Semantic clarity and consistency	Definition	1. Standardized definition of the AOI	Urban centres obtained by the application of the Degree of Urbanisation Method – GHS-SMOD dataset
Thematic and geographic completeness	Coverage	2. Consistent global mapping	Global geographical coverage, 13,135 entries with population ≥50,000 inhabitants in 2015
	Spatial representation	3. Spatially explicit delineation of cities	Geospatial representation with extents at 1 km grid cell resolution (polygons), and centroid XY location (point)
	Spatial, temporal and thematic resolution	4. multi-thematic, multi-dimensional, and multi-temporal attributes	Variables across five thematic domains: geography, socio-economic, environmental, disaster risk reduction, and sustainable development goals. Temporal domain spanning 1975–2015
	Usability	5. comparability of information in space and time	Thematic, geographical and semantic consistency

Existing datasets on cities are few, limited in scope (geographical coverage and biased towards the largest settlements) and thematic contents, and differ by city definition adopted. The main source of urban data to date is the UN World Urbanization Prospects (WUP), which includes 1,860 entries^5^ based on national definition of cities. The WUP provides population information back to 1950 and until recently, it had periodic updates. The WUP is essentially a demographic database on large settlements (population above 300,000 inhabitants), defined with the generic term ‘urban agglomerations’. These are identified by a name and a location (point coordinates). The WUP database has supported most of the literature on cities across scientific^6,7^ and grey sources^8^, despite its intrinsic limitations^3^, mostly due to the punctual location for each city and to the different national definitions on which it relies.

The Global Rural-Urban Mapping Project (GRUMP) dataset instead adopts a geospatial approach combining remote sensing input data (i.e. night-time lights), with a population grid. However, the reference year is 1995 and the database only provides point locations.

The 2030 Development Agenda and the contemporary societal challenges (from climate change and its impacts, to sustainable urbanisation, and widening inequalities amongst others) require a wealth of data that is multi-thematic, reliable, planetary and multi-temporal in coverage, fine scale in detail, open and free in access and, most of all, consistently comparable across space and time^9,10^. All these characteristics require knowledge of the spatial extent of cities, based on a common definition, and the harmonised integration of several data sources, essential information missing in existing databases.

Geospatial data production has surged in recent years, facilitated by the changes towards open and free data policies by long-term Earth observation programmes, such as Landsat, and the launch of new programmes such as European Union Copernicus. Such programmes serve as independent and evidence-based sources of global, continuous and high-resolution information about planet Earth systems and human presence signatures (i.e. settlements, night-time light, emissions, etc.).

The Global Human Settlement Layer project of the European Commission’s Directorate General Joint Research Centre uses big data from Earth Observation and other open geospatial information to produce global spatial information about the human presence on the planet over time^11^, mainly as built-up area (GHS-BUILT), population (GHS-POP) and settlement classification (GHS-SMOD) grids.

In this data descriptor, we present the Global Human Settlement Urban Centre Database (GHS-UCDB^12^). We developed the GHS-UCDB^12^ solutions to solve the gaps of existing databases of cities by implementing GHSL core principles in the production of enriched data features, as shown in Table 1.

In particular, for the definition of cities we rely on the Degree of Urbanisation method^13^ to delineate the areas of interest (AOI) –the urban centres as outlined in the GHS-SMOD layer. For what concerns the thematic and geographic completeness (coverage; spatial representation; spatial, temporal and thematic resolution; usability), we deploy an open geospatial data integration approach.

These production chains aimed at overcoming most of the limitations of available city databases. By following these principles, the GHS-UCDB^12^ integrates a common people-based city definition, thus enhancing temporal and spatial comparisons; addresses full global coverage; provides a spatially explicit characterisation of each city (location –point, and extent –polygon); and incorporates several multi-temporal thematic variables across five thematic domains (geography, socio-economic, environmental, disaster risk reduction, and sustainable development goals).

The resulting dataset is a geospatial database characterising 13,135 urban centres with 160 attributes providing actionable information sourced from open and free data to feed research across several thematic domain of high societal salience, especially in the context of the 2030 Development Agenda and other contemporary societal challenges.

**Fig. 1:**
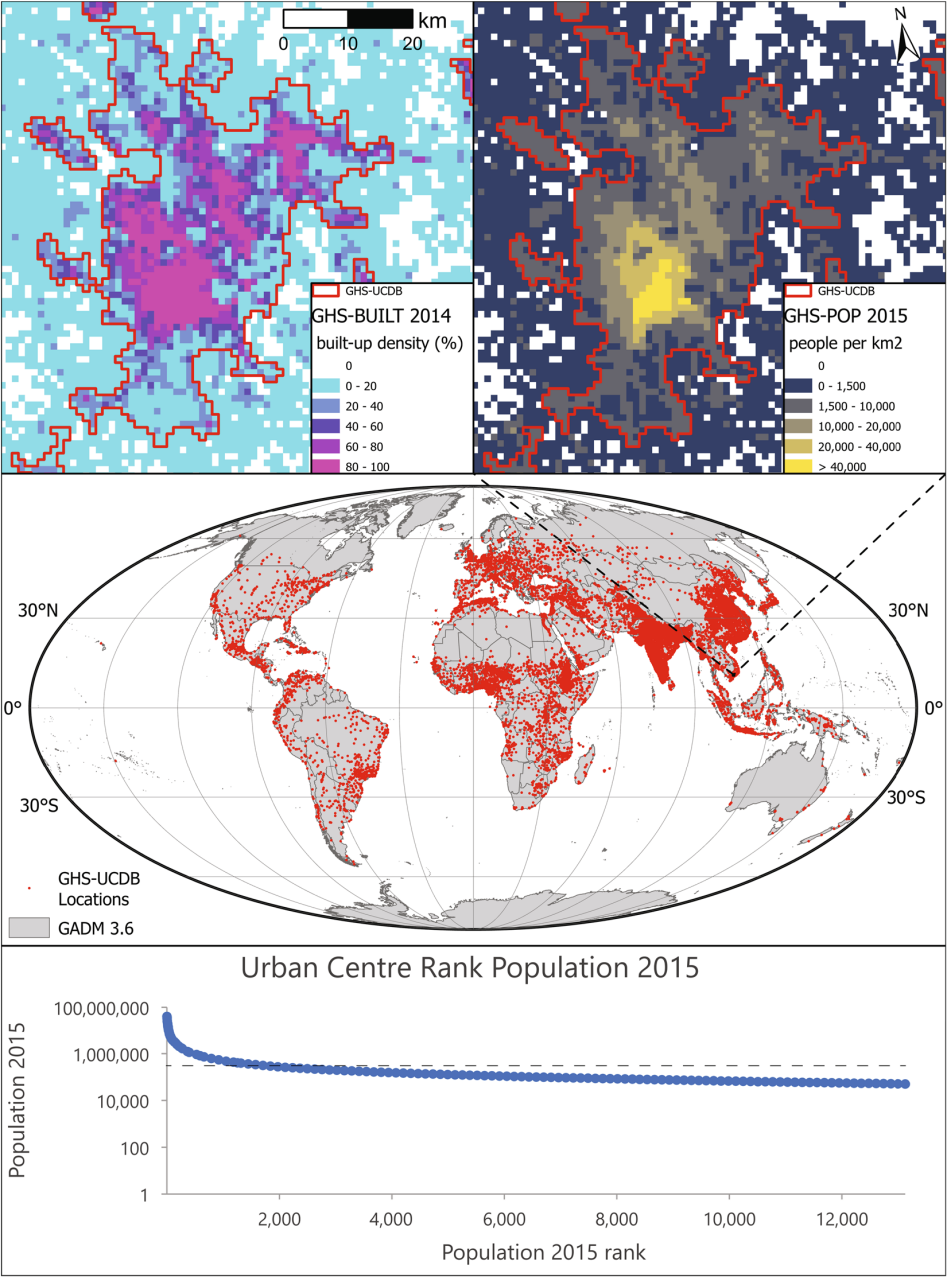
GHS-UCDB distribution and characteristics. The central panel shows the distribution of urban centres (red dots) across the globe (World Mollweide projection). The upper panels show as example the urban centre of Ho Chi Minh in Vietnam and few surrounding urban centres with their boundaries (red solid line) and the layers used for their delineation: the GHS-POP (R2019) population layer at 1 km^2^ resolution for the year 2015 (right panel) and the GHS-BUILT (R2018) built-up area layer at 1 km^2^ resolution for the year 2014. The bottom panel shows the 2015 population of each urban centre in the database (GHS-UCDB) and their rank on the X-axis. The dashed grey line represents the population size lower-bound of the WUP 2018 (300,000 inhabitants). All dots below the dashed line are urban centres that increase the universe of known cities of the world.

As introduced in previous studies^10^, it is possible to use directly GHS-UCDB^12^ variables for analysis purposes such as those resulting in studies on greenness^14^, public health^15^, city demography^16^, urban sprawl^17^, urbanisation^18^ and many others. However, researchers can adopt the *urban centres* AOI for modelling purposes, and produce additional variables for these areas. The GHS-UCDB^12^ has already supported analyses on location and accessibility^19–21^, global studies on anthropogenic and pollutant emissions in urban centres^22,23^, on exposure to extreme heat or to other natural hazards^24,25^. This practice could open the perspective for a community-based update of the dataset where crowd-produced variables are then integrated in the updates of the dataset (Fig. 1).

## Methods

The GHS-UCDB^12^ stands on two main processes: the delineation of the urban centres (the AOI), and the characterisation of the urban centres with multi-thematic and multi-temporal variables.

**Fig. 2:**
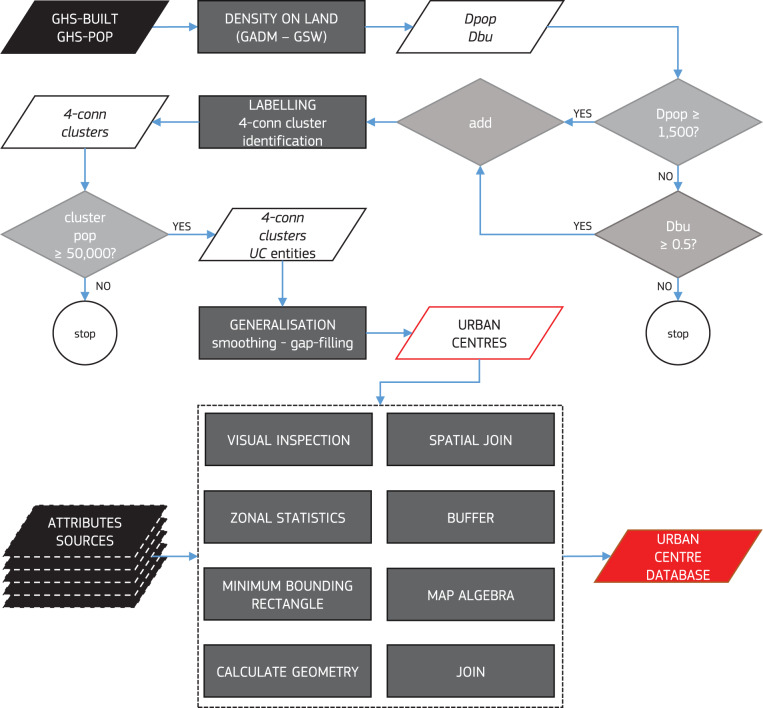
Logical workflow for the delineation of urban centres spatial entities by applying to raster grids the population density, size and grid cell contiguity rules of the Degree of Urbanisation method.

The urban centres are delineated from the settlement classification defined by the Degree of Urbanisation. The Degree of Urbanisation is a method endorsed by the United Nations Statistical Commission to harmonise the collection of international statistics on urban and rural areas^26^. The Degree of Urbanisation method is applied to a 1 km^2^ population grid as a geospatial concept based on population density and size, and grid cells contiguity^13^ with smoothed boundaries and gaps filled (urban centre delineation in Fig. 2). This is translated into a logical definition at the 1 km^2^ grid cell such as:

$$((({D}_{pop}\vee {D}_{bu})\,\varLambda {T}_{con})\,\varLambda {P}_{min})\,\varLambda \,[iterative\_median\_filter\,(3 \mbox{-} by \mbox{-} 3)]\,\varLambda \,[gap\_\,fill]$$

Where *∨* and *Λ* represent the logical “or” and “and” respectively, *D*_*pop*_ is the local (i.e. grid cell) population density lower bound, equal to 1,500 people/km^2^ of permanent land; *D*_*bu*_ is the local share of built-up area on permanent land lower bound, equal to 0.5; *T*_*con*_ is the topological constraint to form clusters with cells respecting the “*Dpop ∨ Dbu*’’ constraint on local population density or local share of built-up area, set to 4-connectivity (i.e. contiguity of cells only along edges, no diagonal adjacency of cells); *P*_*min*_ is the cluster population size lower bound equal to 50,000 people; *iterative_median_filter* is the smoothing applied by 3 × 3 km kernel until idempotence is reached (only additive median filtering); and *gap_fill* is the filling of holes within the urban centre perimeter after the smoothing. Such gaps are filled if they are smaller than 15 km^2^ in surface as described in Florczyk *et al*.^11^. Therefore, urban centres are clusters of contiguous high-density cells (with at least 1,500 people per km^2^ or at least 0.5 km^2^ of built-up area) which altogether contain at least 50,000 inhabitants with smoothed boundaries and gaps below 15 km^2^ filled^13^. The urban centres are extracted from the 2015 GHS-SMOD layer^11^ that implements the Degree of Urbanisation method applied to GHSL layers GHS-BUILT and GHS-POP datasets^27^ of 2015. Additional input data are a refinement of the Database of Global Administrative Areas (GADM2.8) with Global Surface Water (GSW) layer^28^ to determine the permanent land surface per pixel for the computation of densities –on land (see Florczyk *et al*.^29^ for details).

The other process, the characterisation of the urban centres with multi-thematic and multi-temporal variables (geospatial data integration in Fig. 2), is produced in GIS (Geographic Information System) environment by geospatial data integration. Geospatial data across the different thematic dimensions (characteristics, multi-temporal urban centre spatial domain, geography, socio-economic, environment, Disaster Risk Reduction and Sustainable Development Goals) are linked to urban centres entities mainly via GIS operations. Most of the attributes are generated with zonal statistics (i.e. sum or average of source data within each urban centre spatial extent). For example, all the attributes in the socio-economic dimension, and most of the ones in the environment dimension are obtained by means of zonal statistics procedures of raster grids containing population counts, night time light emission, gross domestic product, accessibility, and emissions data (as input value raster) within the urban centre extent (as zone field). In most cases, the spatial data that have different specifications (mainly map projection and resolution) are processed by rasterization and then aligned to the attribute raster grid, afterwards the above mentioned operations are performed. Other variables (especially in the characteristics dimension) are produced with spatial join operations. For example, most of the location variables such as country, geographic region, biome type are obtained using the urban centres as target features, and the spatial delineation of the attribute as join features. Other methods for data integration included map algebra mean for attributes in the geography domain (elevation and precipitation). Variables derived from other attributes in the database (like built-up area per capita) were produced with simple algebraic expressions like ratios directly on the source attributes. Quality control attributes, were obtained by direct visual inspection of urban centres delineation overlaid on very high-resolution satellite imagery by at least three experts per each urban centre entity.

The technical description of the GHS-UCDB^12^ production is contained in the JRC scientific information systems and database report “Description of the GHSL Urban Centre Database 2015”^29^. This data descriptor refines and systematize the production of the 160 attributes for each of the 13,135 urban centres in the database. The Supplementary information to this descriptor organizes the GHS-UCDB^12^ attributes with concise information about the temporal coverage of the database variables, the GIS processing (method) applied to generate each of the attributes (each GHS-UCDB attribute field), and the various input data and corresponding references used to source these variables.

## Data Records

The GHS-UCDB v1.2^12^ is freely available for download via the JRC Open Data Catalogue, the official repository for JRC datasets (https://data.jrc.ec.europa.eu/dataset/53473144-b88c-44bc-b4a3-4583ed1f547e), or GHSL website (https://ghsl.jrc.ec.europa.eu/ghs_stat_ucdb2015mt_r2019a.php). Moreover, the GHS-UCDB can be visually explored online on the GHSL web visualisation portal (https://ghsl.jrc.ec.europa.eu/ucdb2018visual.php).

This dataset is made of a geospatial database compatible with GIS, and a tabular dataset. The geospatial dataset contains a polygon vector layer delineating the boundaries of urban centres (GHS_STAT_UCDB2015MT_GLOBE_R2019A_V1_2.gpkg) and a point vector layer identifying the urban centre centroids (GHS_STAT_UCDB2015MT_GLOBE_R2019A_V1_2_short_pnt.gpkg). Both layers are issued in WGS 84 coordinate system and contain information for 13,135 data records (urban centres). The polygon layer is associated to the complete list of attributes (160 fields) stored in the attribute table, while the point layer has a shorter attribute table with only the main fields (i.e. validation, coordinates, area, country and urban centre main name) – the use can anytime relate the full attribute table to the point dataset via the unique identifier attribute. The complete attribute table (160 attributes) is provided also as tabular data without spatial georeference as excel table (GHS_STAT_UCDB2015MT_GLOBE_R2019A_V1_2.xls) and comma separated value table (GHS_STAT_UCDB2015MT_GLOBE_R2019A_V1_2.csv), while the “short” version only as comma separated value table (GHS_STAT_UCDB2015MT_GLOBE_R2019A_V1_2_short.csv). Most of the variables (Table 2) have multi-temporal coverage (1975 – 2015). Table 2 provides an overview of UCDB attributes by thematic area and temporal coverage. The epoch for non-time-continuous/complete datasets has been selected in a way to approximate the closest time point to the year 1975-1990-2000-2015. For example, the attribute Maximum magnitude of the heatwave (EX_HW_IDX) covers 1980 to 2010 as no data was available for 1975 and 2015. On the contrary, the richness of attributes in the time series was preserved, where available. For example, the attribute E_CPM2_*xx* (Total concertation of PM_2.5_) has no data prior to 2000, but it has a richer time series afterwards and includes 2000, 2005, 2010 and 2014. The Supplementary information lists all attributes with their respective temporal coverage and indicates the specific year. Florczyk *et al*.^29^ provides a detailed description of the GHS-UCDB v1, yet the continuous improvement and refinement of the database was consolidated into the GHS-UCDB v1.2. This latest version includes an update to the following attributes: UC_NM_MN, UC_NM_LST, UC_NM_SRC referring to the naming of urban centres according to the workflow explained in Florczyk *et al*.^11^ (based new inputs –GISCO and Open Streets Map databases, and algorithm –priority filtering based on buffered spatial join); TT2CC, the travel time to country capital; E_GR_AV90, E_GR_AV00, E_GR_AV14 fields reporting the average greenness of the urban centres in 1990, 2000, and 2014. Moreover, all attributes related to emissions were aligned to the epoch 2015 (2012 in the GHS-UCDB v1) leveraging on the new edition of the EDGAR database (v5)^30^. Eventual revisions of the dataset v1.2 will be documented in the changelog on the GHSL Website –see Changelog v1.2 (https://ghsl.jrc.ec.europa.eu/ghs_stat_ucdb2015mt_r2019a.php).

**Table 2:** Summary of GHS-UCDB dimensions, variables and temporal coverage.

Dimensions	Variable	Temporal coverage			
General characteristics	Identification	2015			
	Extension				
	Location				
	Name				
Multi-temporal urban centre spatial domain	Number	1975	1990	2000	2015
	Area				
Geography	Elevation	2015			
	Biome				
	Climate	1986–2010			
	Soil	2015			
	River basin				
	Temperature			2000	2015
	Precipitation				
Socio-economic	Resident population	1975	1990	2000	2015
	Built-up area				
	Night time light	2015			
	GDP		1990	2000	2015
	Development	2018			
	Accessibility & remoteness	2015			
Environment	Greenness		1990	2000	2015
	Pollutants’ emission	1975	1990	2000	2015
	Pollutants’ concentration	2000	2005	2010	2015
DRR	Floods (exposure)	1975	1990	2000	2015
	Earthquake (exposure)				
	Storm surge (exposure)				
	Maximum magnitude of heatwaves	1980–2010			
SDG	Land Use Efficiency (11.3.1)	1990–2015			
	Open spaces (11.7.1 –proxy)	2015			

## Technical Validation

Technical validation of the GHS-UCDB^12^ was performed by direct visual inspection of the 13,135 urban centre polygons. To do so, at least three interpreters assessed the presence of human settlements with very-high resolution satellite imagery (Google or Bing imagery). The validation was performed via GIS interface overlaying the outlines of urban centres to satellite imagery. Each of the interpreters assigned an individual quality score. Combined scores resulted in the three “QA2_1V” field values: 0- false positive (no presence of high-density human settlement); 1- true positive (confirmed presence of high-density human settlement); 2- uncertain (disagreement between experts, or presence of high-density settlement unclear). The breakdown of urban centres by quality assessment is: 258 false positives (2% of the sample), 2,574 uncertain (20%); 10,303 true positives (78%). Based on the only spatial attribute in the UNDESA WUP we verified that all the 1,860 entries in the WUP database (WUP2018-F12-Cities_Over_300K.xls) do spatially match with an urban centre, and therefore: 644 UC match a city proper, 178 a metropolitan area, and 1,038 an urban agglomeration.

Other variables in the database are externally sourced from existing authoritative data sources as best available at time of production of UCDB. These are taken to be of sufficient robustness and quality for the purpose, and their production and validation is documented in the respective peer reviewed publications. Despite efforts in ensuring the fitness for purpose of input data, thematic variables were not subject to technical validation in the frame of our work, as these data were not directly produced by the authors. Such information can be retrieved from their respective publications.

## Usage Notes

The use of GHS-UCDB can be facilitated filtering urban centres by the quality assessment attribute to ensure the highest data quality possible, while still maintaining a large geographical coverage. The designations employed and the presentation of materials and maps do not imply the expression of any opinion whatsoever on the part of the European Union concerning the legal status of any country, territory or area or of its authorities, or concerning the delimitation of its frontiers or boundaries that if shown on the maps are only indicative. The boundaries and names shown on maps and databases do not imply official endorsement or acceptance by the European Union. The views expressed herein are those of the authors and do not necessarily reflect the views of the European Union.

### Supplementary information

Supplementary material

## Data Availability

The GHSL project produces free and open tools such as the GHS-DUG^31^ tool (https://ghsl.jrc.ec.europa.eu/tools.php) that outputs the settlement classification, by applying the Degree of Urbanisation to input population and built-up area grids. One of the outputs of this tool is the shapefile urban centres delineations that are the spatial units used in this study.
