# Supplementary material for: The Multi-temporal and Multi-dimensional Global Urban Centre Database to Delineate and Analyse World Cities
Source: Sci Data. 2024 Jan 17;11:82. doi: 10.1038/s41597-023-02691-1 (PMC10794220; doi:10.1038/s41597-023-02691-1)
Supplement: Supplementary file 1 — Supplementary material [file 41597_2023_2691_MOESM1_ESM.docx]

**The Multi-temporal and Multi-dimensional Global Urban Centre Database to Delineate and Analyse World Cities**

### Authors

Michele Melchiorri^1^, Sergio Freire^1^, Marcello Schiavina^1^, Aneta Florczyk^1^, Christina Corbane^1^, Luca Maffenini^2^, Martino Pesaresi^1^, Panagiotis Politis^3^, Filip Szabo^1^, Daniele Ehrlich^1^, Pierpaolo Tommasi^5^, Donato Airaghi^6^, Luigi Zanchetta^1^, Thomas Kemper^1^

**Affiliations**

1. European Commission, Joint Research Centre, Ispra, Italy

2. Uni Systems, Luxembourg, Luxembourg

3. Arhs Developments S.A., Belvaux, Luxembourg

corresponding author: Michele Melchiorri (michele.melchiorri@ec.europa.eu)

### Supplementary material

Schematic description of the GHS-UCDB attributes with concise information about the temporal coverage of the database variables, the GIS processing (method) applied to generate each of the attributes (each GHS-UCDB attribute field), and the various input data and corresponding references used to source these variables.

Supplementary Table 1 - Variables and attributes in the general characteristics dimension

| **Dimension** | **Variable** | **Temporal coverage** | | | | **Attribute** | **GHS-UCDB** | **Metric** | **Input data** | **Method** | **Note** |
| --- | --- | --- | --- | --- | --- | --- | --- | --- | --- | --- | --- |
|  |  | **1975** | **1990** | **2000** | **2015** |  | **Field** |  |  |  |  |
|  | Control code |  |  |  | ■ | Unique ID | ID_HDC_G0 |  |  |  |  |
| **characteristics** |  |  |  |  | ■ | Quality Code | QA2_1V |  | Florczyk et al., 2018 | Visual inspection | Class (1) |
|  | Extension |  |  |  | ■ | Area | AREA | km^2^ | Derived | Zonal statistics (sum) |  |
|  |  |  |  |  | ■ | Bounding Box (WGS 84) | BBX_LATMN, | ° | Derived | Minimum Bounding Rectangle tool | Decimal Degrees |
|  |  |  |  |  |  |  | BBX_LONMN, |  |  |  |  |
|  |  |  |  |  |  |  | BBX_LATMX, |  |  |  |  |
|  |  |  |  |  |  |  | BBX_LONMX |  |  |  |  |
|  | Location |  |  |  | ■ | Geometric Centroid (WGS 84) | GCPNT_LAT, | ° | Derived | Calculate geometry | Decimal Degrees |
|  |  |  |  |  |  |  | GCPNT_LON |  |  |  |  |
|  |  |  |  |  | ■ | Main Country Identification: name | CTR_MN_NM |  | (GADM, 2018) | Spatial join (feature) |  |
|  |  |  |  |  | ■ | Main Country Identification: ISO 3 | CTR_MN_ISO |  | (GADM, 2018) |  |  |
|  |  |  |  |  | ■ | Cross border flag | XBRDR |  | JRC |  | Boolean |
|  |  |  |  |  | ■ | Number of intersected countries | XCTR_NBR |  | JRC |  | Number of entities. |
|  |  |  |  |  | ■ | List of intersected countries: names | XC_NM_LST |  | (GADM, 2018) |  |  |
|  |  |  |  |  | ■ | List of intersected countries: ISO 3 codes | XC_ISO_LST |  | (GADM, 2018) |  |  |
|  |  |  |  |  | ■ | Major Geographical Region | GRGN_L1 |  | UN WUP 2018 |  | Class (2) |
|  |  |  |  |  | ■ | Geographical Region | GRGN_L2 |  | UN WUP 2018 |  | Class (3) |
|  | Name |  |  |  | ■ | Name of the Urban Centre | UC_NM_MN |  | GISCO, OSM, and Florczyk et al. 2019 | Spatial join |  |
|  |  |  |  |  | ■ | List of names | UC_NM_LST |  |  | (feature, 3 km buffer) |  |
|  |  |  |  |  | ■ | Source of the names | UC_NM_SRC |  |  |  | Class (4) |

**Supplementary Table 1 continued - Variables and attributes in the general characteristics dimension**

| **Dimension** | **Variable** | **Temporal coverage** | | | | **Attribute** | **GHS-UCDB** | **Metric** | **Input data** | **Method** | **Note** |
| --- | --- | --- | --- | --- | --- | --- | --- | --- | --- | --- | --- |
|  |  | **1975** | **1990** | **2000** | **2015** |  | **Field** |  |  |  |  |
| **Multi-temporal domain** | Number of Urban Centres in the past | ■ | ■ | ■ |  | Number of Urban Centres in 1975 | H75_NBR |  | (Florczyk et al., 2018) |  | Number of entities |
|  |  |  |  |  |  | Number of Urban Centres in 1990 | H90_NBR |  |  |  | Number of entities |
|  |  |  |  |  |  | Number of Urban Centres in 2000 | H00_NBR |  |  |  | Number of entities |
|  | Total area of Urban Centres in the past | ■ | ■ | ■ |  | Total area of Urban Centres in 1975 | H75_AREA | km^2^ |  | Zonal statistics (sum) |  |
|  |  |  |  |  |  | Total area of Urban Centres in 1990 | H90_AREA | km^2^ |  |  |  |
|  |  |  |  |  |  | Total area of Urban Centres in 2000 | H00_AREA | km^2^ |  |  |  |

Supplementary Table 2 - Variables and attributes in the geography dimension

| **Dimension** | **Variable** | **Temporal coverage** | | | | **Attribute** | **GHS-UCDB** | **Metric** | **Input data** | **Method** | **Note** |
| --- | --- | --- | --- | --- | --- | --- | --- | --- | --- | --- | --- |
|  |  | **1975** | **1990** | **2000** | **2015** |  | **Field** |  |  |  |  |
| Geography | Biome |  |  |  | ■ | Biome type(s) | E_BM_NM_LST |  | (Olson et al. 2001) | Spatial join (feature) | Class (5) |
|  | Soil |  |  |  | ■ | Soil group(s) | E_SL_LST |  | (Fischer et al. 2008) |  | Class (6) |
|  | Elevation |  |  |  | ■ | Average Elevation | EL_AV_ALS | m | (Tadono et al. 2016) | Map algebra (mean) | Meters above sea level (MASL) |
|  | Climate | ■ | | | | Climate class(es) | E_KG_NM_LST |  | (Rubel et al. 2017) | Spatial join (feature) | Class (7) |
|  |  | 1986-2010 | | | |  |  |  |  |  |  |
|  | River basin |  |  |  | ■ | Major river basin(s) | E_RB_NM_LST |  | (Global Runoff Data Centre 2007) |  | Class (8) |
|  | Precipitation |  | ■ | ■ | ■ | Average precipitation for epoch 1990 | E_WR_P_90 | mm | (Harris et al., 2014) | Map algebra (mean) |  |
|  |  |  |  |  |  | Average precipitation for epoch 2000 | E_WR_P_00 | mm |  |  |  |
|  |  |  |  |  |  | Average precipitation for epoch 2014 | E_WR_P_14 | mm |  |  |  |
|  | Temperature |  | ■ | ■ | ■ | Average temperature for epoch 1990 | E_WR_T_90 | °C |  |  |  |
|  |  |  |  |  |  | Average temperature for epoch 2000 | E_WR_T_00 | °C |  |  |  |
|  |  |  |  |  |  | Average temperature for epoch 2014 | E_WR_T_14 | °C |  |  |  |

Supplementary Table 3 - Variables and attributes in the socio-economic dimension

| **Dimension** | **Variable** | **Temporal coverage** | | | | **Attribute** | **GHS-UCDB** | **Metric** | **Input data** | **Method** | **Note** |
| --- | --- | --- | --- | --- | --- | --- | --- | --- | --- | --- | --- |
|  |  | **1975** | **1990** | **2000** | **2015** |  | **Field** |  |  |  |  |
| **socio-economic** | Built-up surface | ■ | ■ | ■ | ■ | Total built-up area in 1975 | B75 | km^2^ | GHS_BUILT_LDS1975 | Zonal statistics (sum) |  |
|  |  |  |  |  |  |  |  |  | _GLOBE_R2018A |  |  |
|  |  |  |  |  |  |  |  |  | _54009_1K_V_1_0 (Florczyk et al., 2018) |  |  |
|  |  |  |  |  |  | Total built-up area in 1990 | B90 | km^2^ | GHS_BUILT_LDS1990 |  |  |
|  |  |  |  |  |  |  |  |  | _GLOBE_R2018A |  |  |
|  |  |  |  |  |  |  |  |  | _54009_1K_V_1_0 (Florczyk et al., 2018) |  |  |
|  |  |  |  |  |  | Total built-up area in 2000 | B00 | km^2^ | GHS_BUILT_LDS2000 |  |  |
|  |  |  |  |  |  |  |  |  | _GLOBE_R2018A |  |  |
|  |  |  |  |  |  |  |  |  | _54009_1K_V_1_0 (Florczyk et al., 2018) |  |  |
|  |  |  |  |  |  | Total built-up area in 2015 | B15 | km^2^ | GHS_BUILT_LDS2015 |  |  |
|  |  |  |  |  |  |  |  |  | _GLOBE_R2018A |  |  |
|  |  |  |  |  |  |  |  |  | _54009_1K_V_1_0 (Florczyk et al., 2018) |  |  |
|  | Resident population | ■ | ■ | ■ | ■ | Total resident population in 1975 | P75 |  | GHS_POP_GPW41E1975 | Zonal statistics (sum) | Number of people |
|  |  |  |  |  |  |  |  |  | _GLOBE_R2018A |  |  |
|  |  |  |  |  |  |  |  |  | _54009_1K_V_1_0 (Florczyk et al., 2018) |  |  |
|  |  |  |  |  |  | Total resident population in 1990 | P90 |  | GHS_POP_GPW41E1990 |  | Number of people |
|  |  |  |  |  |  |  |  |  | _GLOBE_R2018A |  |  |
|  |  |  |  |  |  |  |  |  | _54009_1K_V_1_0 (Florczyk et al., 2018) |  |  |
|  |  |  |  |  |  | Total resident population in 2000 | P00 |  | GHS_POP_GPW41E2000 |  | Number of people |
|  |  |  |  |  |  |  |  |  | _GLOBE_R2018A |  |  |
|  |  |  |  |  |  |  |  |  | _54009_1K_V_1_0 (Florczyk et al., 2018) |  |  |
|  |  |  |  |  |  | Total resident population in 2015 | P15 |  | GHS_POP_GPW41E2015 |  | Number of people |
|  |  |  |  |  |  |  |  |  | _GLOBE_R2018A |  |  |
|  |  |  |  |  |  |  |  |  | _54009_1K_V_1_0 (Florczyk et al., 2018) |  |  |
|  | Built-up per capita | ■ | ■ | ■ | ■ | Surface of the built-up area per person in 1975 | BUCAP75 | m^2^ person^-1^ | Derived | ratio | Sq m per person |
|  |  |  |  |  |  | Surface of the built-up area per person in 1990 | BUCAP90 | m^2^ person^-1^ | Derived |  | Sq m per person |
|  |  |  |  |  |  | Surface of the built-up area per person in 2000 | BUCAP00 | m^2^ person^-1^ | Derived |  | Sq m per person |
|  |  |  |  |  |  | Surface of the built-up area per person in 2015 | BUCAP15 | m^2^ person^-1^ | Derived |  | Sq m per person |
|  | Night time light emission |  |  |  | ■ | Average night time light emission in 2015 | NTL_AV | nW cm^-2^ sr^-1^ | (Weiss et al., 2018) | Zonal statistics (average) | nano-watt per steradian per square centimetre |
|  | Gross Domestic Product |  | ■ | ■ | ■ | Sum of GDP PPP values for year 1990 | GDP90_SM | $ | (Kummu et al., 2018) | Zonal statistics (sum) | USA dollar 2011 |
|  |  |  |  |  |  | Sum of GDP PPP values for year 2000 | GDP00_SM | $ |  |  | USA dollar 2011 |
|  |  |  |  |  |  | Sum of GDP PPP values for year 2015 | GDP15_SM | $ |  |  | USA dollar 2011 |
|  | Development Indicators |  |  |  | ■ | UN income class | INCM_CMI |  | (UNDESA, 2018b) | join | Class (9) |
|  |  |  |  |  | ■ | UN development group | DEV_CMI |  |  |  | Class (10) |
|  | Accessibility & Remoteness |  |  |  | ■ | Travel time to country capital | TT2CC | ’ | (Weiss et al., 2018), JRC | Zonal statistics (sum) | minutes |

Supplementary Table 4 - Variables and attributes in the environment dimension

| **Dimension** | **Variable** | **Temporal coverage** | | | | **Attribute** | | **GHS-UCDB** | **Metric** | **Input data** | **Method** | **Note** |
| --- | --- | --- | --- | --- | --- | --- | --- | --- | --- | --- | --- | --- |
|  |  | **1975** | **1990** | **2000** | **2015** |  |  | **Field** |  |  |  |  |
|  | Urban green (NDVI) |  | ■ | ■ | ■ | **Greenness Estimate** | Average greenness estimated for 1990 located in the built-up area of epoch 1990 | E_GR_AV90 |  | (Corbane et al., 2018b) | Zonal statistics (average) | Index |
|  |  |  |  |  |  |  | Average greenness estimated for 2000 located in the built-up area of epoch 2000 | E_GR_AV00 |  |  |  | Index |
| **Environment** |  |  |  |  |  |  | Average greenness estimated for 2014 located in the built-up area of epoch 2014 | E_GR_AV14 |  |  |  | Index |
|  | Urban green (class area) |  | ■ | ■ | ■ | **Greenness class area** | Total area of the high green estimated for 1990 | E_GR_AH90 | km^2^ | (Corbane et al., 2018b) | Zonal statistics (sum) |  |
|  |  |  |  |  |  |  | Total area of the medium green estimated for 1990 | E_GR_AM90 | km^2^ |  |  |  |
|  |  |  |  |  |  |  | Total area of the low green estimated for 1990 | E_GR_AL90 | km^2^ |  |  |  |
|  |  |  |  |  |  |  | Total area of green estimated for 1990 | E_GR_AT90 | km^2^ |  |  |  |
|  |  |  |  |  |  |  | Total area of the high green estimated for 2000 | E_GR_AH00 | km^2^ |  |  |  |
|  |  |  |  |  |  |  | Total area of the medium green estimated for 2000 | E_GR_AM00 | km^2^ |  |  |  |
|  |  |  |  |  |  |  | Total area of the low green estimated for 2000 | E_GR_AL00 | km^2^ |  |  |  |
|  |  |  |  |  |  |  | Total area of green estimated for 2000 | E_GR_AT00 | km^2^ |  |  |  |
|  |  |  |  |  |  |  | Total area of the high green estimated for 2014 | E_GR_AH14 | km^2^ |  |  |  |
|  |  |  |  |  |  |  | Total area of the medium green estimated for 2000 | E_GR_AM14 | km^2^ |  |  |  |
|  |  |  |  |  |  |  | Total area of the low green estimated for 2014 | E_GR_AL14 | km^2^ |  |  |  |
|  |  |  |  |  |  |  | Total area of green estimated for 2014 | E_GR_AT14 | km^2^ |  |  |  |
|  | Total CO_2_ emissions | ■ | ■ | ■ | ■ | **CO_2_ (non-short-cycle-organic fuels)** | Total emission of CO_2_ from the energy sector, using non-short-cycle-organic fuels in 1975 | E_EC2E_E90 | t a^-1^ | (Crippa et al., 2018) |  | tonnes (10^3^ kg) per year |
|  |  |  |  |  |  |  | Total emission of CO_2_ from the energy sector, using non-short-cycle-organic fuels in 1990 | E_EC2E_E90 | t a^-1^ |  |  | tonnes (10^3^ kg) per year |
|  |  |  |  |  |  |  | Total emission of CO_2_ from the energy sector, using non-short-cycle-organic fuels in 2000 | E_EC2E_E00 | t a^-1^ |  |  | tonnes (10^3^ kg) per year |
|  |  |  |  |  |  |  | Total emission of CO_2_ from the energy sector, using non-short-cycle-organic fuels in 2015 | E_EC2E_E15 | t a^-1^ |  |  | tonnes (10^3^ kg) per year |
|  |  |  |  |  |  |  | Total emission of CO_2_ from the residential sector, using non-short-cycle-organic fuels in 1975 | E_EC2E_R75 | t a^-1^ |  |  | tonnes (10^3^ kg) per year |
|  |  |  |  |  |  |  | Total emission of CO_2_ from the residential sector, using non-short-cycle-organic fuels in 1990 | E_EC2E_R90 | t a^-1^ |  |  | tonnes (10^3^ kg) per year |
|  |  |  |  |  |  |  | Total emission of CO_2_ from the residential sector, using non-short-cycle-organic fuels in 2000 | E_EC2E_R00 | t a^-1^ |  |  | tonnes (10^3^ kg) per year |
|  |  |  |  |  |  |  | Total emission of CO_2_ from the residential sector, using non-short-cycle-organic fuels in 2015 | E_EC2E_R15 | t a^-1^ |  |  | tonnes (10^3^ kg) per year |
|  |  |  |  |  |  |  | Total emission of CO_2_ from the industry sector, using non-short-cycle-organic fuels in 1975 | E_EC2E_I75 | t a^-1^ |  |  | tonnes (10^3^ kg) per year |
|  |  |  |  |  |  |  | Total emission of CO_2_ from the industry sector, using non-short-cycle-organic fuels in 1990 | E_EC2E_I90 | t a^-1^ |  |  | tonnes (10^3^ kg) per year |
|  |  |  |  |  |  |  | Total emission of CO_2_ from the industry sector, using non-short-cycle-organic fuels in 2000 | E_EC2E_I00 | t a^-1^ |  |  | tonnes (10^3^ kg) per year |
|  |  |  |  |  |  |  | Total emission of CO_2_ from the industry sector, using non-short-cycle-organic fuels in 2015 | E_EC2E_I15 | t a^-1^ |  |  | tonnes (10^3^ kg) per year |
|  |  |  |  |  |  |  | Total emission of CO_2_ from the transport sector, using non-short-cycle-organic fuels in 1975 | E_EC2E_T75 | t a^-1^ |  |  | tonnes (10^3^ kg) per year |
|  |  |  |  |  |  |  | Total emission of CO_2_ from the transport sector, using non-short-cycle-organic fuels in 1990 | E_EC2E_T90 | t a^-1^ |  |  | tonnes (10^3^ kg) per year |
|  |  |  |  |  |  |  | Total emission of CO_2_ from the transport sector, using non-short-cycle-organic fuels in 2000 | E_EC2E_T00 | t a^-1^ |  |  | tonnes (10^3^ kg) per year |
|  |  |  |  |  |  |  | Total emission of CO_2_ from the transport sector, using non-short-cycle-organic fuels in 2015 | E_EC2E_T15 | t a^-1^ |  |  | tonnes (10^3^ kg) per year |
|  |  |  |  |  |  |  | Total emission of CO_2_ from the agriculture sector, using non-short-cycle-organic fuels in 1975 | E_EC2E_A75 | t a^-1^ |  |  | tonnes (10^3^ kg) per year |
|  |  |  |  |  |  |  | Total emission of CO_2_ from the agriculture sector, using non-short-cycle-organic fuels in 1990 | E_EC2E_A90 | t a^-1^ |  |  | tonnes (10^3^ kg) per year |
|  |  |  |  |  |  |  | Total emission of CO_2_ from the agriculture sector, using non-short-cycle-organic fuels in 2000 | E_EC2E_A00 | t a^-1^ |  |  | tonnes (10^3^ kg) per year |
|  |  |  |  |  |  |  | Total emission of CO_2_ from the agriculture sector, using non-short-cycle-organic fuels in 2015 | E_EC2E_A15 | t a^-1^ |  |  | tonnes (10^3^ kg) per year |
|  |  | ■ | ■ | ■ | ■ | **CO_2_ (short-cycle-organic fuels)** | Total emission of CO_2_ from the energy sector, using short-cycle-organic fuels in 1975 | E_EC2O_E75 | t a^-1^ | (Crippa et al., 2018) |  | tonnes (10^3^ kg) per year |
|  |  |  |  |  |  |  | Total emission of CO_2_ from the energy sector, using short-cycle-organic fuels in 1990 | E_EC2O_E90 | t a^-1^ |  |  | tonnes (10^3^ kg) per year |
|  |  |  |  |  |  |  | Total emission of CO_2_ from the energy sector, using short-cycle-organic fuels in 2000 | E_EC2O_E00 | t a^-1^ |  |  | tonnes (10^3^ kg) per year |
|  |  |  |  |  |  |  | Total emission of CO_2_ from the energy sector, using short-cycle-organic fuels in 2015 | E_EC2O_E15 | t a^-1^ |  |  | tonnes (10^3^ kg) per year |
|  |  |  |  |  |  |  | Total emission of CO_2_ from the residential sector, using short-cycle-organic fuels in 1975 | E_EC2O_R75 | t a^-1^ |  |  | tonnes (10^3^ kg) per year |
|  |  |  |  |  |  |  | Total emission of CO_2_ from the residential sector, using short-cycle-organic fuels in 1990 | E_EC2O_R90 | t a^-1^ |  |  | tonnes (10^3^ kg) per year |
|  |  |  |  |  |  |  | Total emission of CO_2_ from the residential sector, using short-cycle-organic fuels in 2000 | E_EC2O_R00 | t a^-1^ |  |  | tonnes (10^3^ kg) per year |
|  |  |  |  |  |  |  | Total emission of CO_2_ from the residential sector, using short-cycle-organic fuels in 2015 | E_EC2O_R15 | t a^-1^ |  |  | tonnes (10^3^ kg) per year |
|  |  |  |  |  |  |  | Total emission of CO_2_ from the industry sector, using short-cycle-organic fuels in 1975 | E_EC2O_I75 | t a^-1^ |  |  | tonnes (10^3^ kg) per year |
|  |  |  |  |  |  |  | Total emission of CO_2_ from the industry sector, using short-cycle-organic fuels in 1990 | E_EC2O_I90 | t a^-1^ |  |  | tonnes (10^3^ kg) per year |
|  |  |  |  |  |  |  | Total emission of CO_2_ from the industry sector, using short-cycle-organic fuels in 2000 | E_EC2O_I00 | t a^-1^ |  |  | tonnes (10^3^ kg) per year |
|  |  |  |  |  |  |  | Total emission of CO_2_ from the industry sector, using short-cycle-organic fuels in 2015 | E_EC2O_I15 | t a^-1^ |  |  | tonnes (10^3^ kg) per year |
|  |  |  |  |  |  |  | Total emission of CO_2_ from the transport sector, using short-cycle-organic fuels in 1975 | E_EC2O_T75 | t a^-1^ |  |  | tonnes (10^3^ kg) per year |
|  |  |  |  |  |  |  | Total emission of CO_2_ from the transport sector, using short-cycle-organic fuels in 1990 | E_EC2O_T90 | t a^-1^ |  |  | tonnes (10^3^ kg) per year |
|  |  |  |  |  |  |  | Total emission of CO_2_ from the transport sector, using short-cycle-organic fuels in 2000 | E_EC2O_T00 | t a^-1^ |  |  | tonnes (10^3^ kg) per year |
|  |  |  |  |  |  |  | Total emission of CO_2_ from the transport sector, using short-cycle-organic fuels in 2015 | E_EC2O_T15 | t a^-1^ |  |  | tonnes (10^3^ kg) per year |
|  |  |  |  |  |  |  | Total emission of CO_2_ from the agriculture sector, using short-cycle-organic fuels in 1975 | E_EC2O_A75 | t a^-1^ |  |  | tonnes (10^3^ kg) per year |
|  |  |  |  |  |  |  | Total emission of CO_2_ from the agriculture sector, using short-cycle-organic fuels in 1990 | E_EC2O_A90 | t a^-1^ |  |  | tonnes (10^3^ kg) per year |
|  |  |  |  |  |  |  | Total emission of CO_2_ from the agriculture sector, using short-cycle-organic fuels in 2000 | E_EC2O_A00 | t a^-1^ |  |  | tonnes (10^3^ kg) per year |
|  |  |  |  |  |  |  | Total emission of CO_2_ from the agriculture sector, using short-cycle-organic fuels in 2015 | E_EC2O_A15 | t a^-1^ |  |  | tonnes (10^3^ kg) per year |
|  | Total PM_2.5_ emissions | ■ | ■ | ■ | ■ | **PM_2.5_** | Total emission of PM_2.5_ from the energy sector in 1975 | E_EPM2_E75 | t a^-1^ | (Crippa et al., 2018) |  | tonnes (10^3^ kg) per year |
|  |  |  |  |  |  |  | Total emission of PM_2.5_ from the energy sector in 1990 | E_EPM2_E90 | t a^-1^ |  |  | tonnes (10^3^ kg) per year |
|  |  |  |  |  |  |  | Total emission of PM_2.5_ from the energy sector in 2000 | E_EPM2_E00 | t a^-1^ |  |  | tonnes (10^3^ kg) per year |
|  |  |  |  |  |  |  | Total emission of PM_2.5_ from the energy sector in 2015 | E_EPM2_E15 | t a^-1^ |  |  | tonnes (10^3^ kg) per year |
|  |  |  |  |  |  |  | Total emission of PM_2.5_ from the residential sector in 1975 | E_EPM2_R75 | t a^-1^ |  |  | tonnes (10^3^ kg) per year |
|  |  |  |  |  |  |  | Total emission of PM_2.5_ from the residential sector in 1990 | E_EPM2_R90 | t a^-1^ |  |  | tonnes (10^3^ kg) per year |
|  |  |  |  |  |  |  | Total emission of PM_2.5_ from the residential sector in 2000 | E_EPM2_R00 | t a^-1^ |  |  | tonnes (10^3^ kg) per year |
|  |  |  |  |  |  |  | Total emission of PM_2.5_ from the residential sector in 2015 | E_EPM2_R15 | t a^-1^ |  |  | tonnes (10^3^ kg) per year |
|  |  |  |  |  |  |  | Total emission of PM_2.5_ from the industry sector in 1975 | E_EPM2_I75 | t a^-1^ |  |  | tonnes (10^3^ kg) per year |
|  |  |  |  |  |  |  | Total emission of PM_2.5_ from the industry sector in 1990 | E_EPM2_I90 | t a^-1^ |  |  | tonnes (10^3^ kg) per year |
|  |  |  |  |  |  |  | Total emission of PM_2.5_ from the industry sector in 2000 | E_EPM2_I00 | t a^-1^ |  |  | tonnes (10^3^ kg) per year |
|  |  |  |  |  |  |  | Total emission of PM_2.5_ from the industry sector in 2015 | E_EPM2_I15 | t a^-1^ |  |  | tonnes (10^3^ kg) per year |
|  |  |  |  |  |  |  | Total emission of PM_2.5_ from the transport sector in 1975 | E_EPM2_T75 | t a^-1^ |  |  | tonnes (10^3^ kg) per year |
|  |  |  |  |  |  |  | Total emission of PM_2.5_ from the transport sector in 1990 | E_EPM2_T90 | t a^-1^ |  |  | tonnes (10^3^ kg) per year |
|  |  |  |  |  |  |  | Total emission of PM_2.5_ from the transport sector in 2000 | E_EPM2_T00 | t a^-1^ |  |  | tonnes (10^3^ kg) per year |
|  |  |  |  |  |  |  | Total emission of PM_2.5_ from the transport sector in 2015 | E_EPM2_T15 | t a^-1^ |  |  | tonnes (10^3^ kg) per year |
|  |  |  |  |  |  |  | Total emission of PM2_2.5_ from the agriculture sector in 1975 | E_EPM2_A75 | t a^-1^ |  |  | tonnes (10^3^ kg) per year |
|  |  |  |  |  |  |  | Total emission of PM_2.5_ from the agriculture sector in 1990 | E_EPM2_A90 | t a^-1^ |  |  | tonnes (10^3^ kg) per year |
|  |  |  |  |  |  |  | Total emission of PM_2.5_ from the agriculture sector in 2000 | E_EPM2_A00 | t a^-1^ |  |  | tonnes (10^3^ kg) per year |
|  |  |  |  |  |  |  | Total emission of PM_2.5_ from the agriculture sector in 2015 | E_EPM2_A15 | t a^-1^ |  |  | tonnes (10^3^ kg) per year |
|  | Concertation of Pollutants |  | ■ | | | **PM_2.5_** | Total concertation of PM_2.5_ for reference epoch 2000 | E_CPM2_T00 | μg m^-3^ | (Global Burden of Disease Collaborative Network 2018),(OECD Environment Statistics (database) 2018) | Zonal statistics (average) | micrograms per cubic meter air |
|  |  |  | 2000 | | |  |  |  |  |  |  |  |
|  |  |  | 2005 | | |  | Total concertation of PM_2.5_ for reference epoch 2005 | E_CPM2_T05 | μg m^-3^ |  |  | micrograms per cubic meter air |
|  |  |  | 2010 | | |  | Total concertation of PM_2.5_ for reference epoch 2010 | E_CPM2_T10 | μg m^-3^ |  |  | micrograms per cubic meter air |
|  |  |  | 2014 | | |  | Total concertation of PM_2.5_ for reference epoch 2014 | E_CPM2_T14 | μg m^-3^ |  |  | micrograms per cubic meter air |

Supplementary Table 5 - Variables and attributes in the hazards and exposure (DRR) dimension

| **Dimension** | **Variable** | **Temporal coverage** | | | | **Attribute** | **GHS-UCDB** | **Metric** | **Input data** | **Method** | **Note** |
| --- | --- | --- | --- | --- | --- | --- | --- | --- | --- | --- | --- |
|  |  | **1975** | **1990** | **2000** | **2015** |  | **Field** |  |  |  |  |
|  | Flood exposure |  |  |  | ■ | Total surface potentially exposed to floods | EX_FD_AREA | km^2^ | (Dottori et al. 2016) | Zonal statistics (sum) |  |
|  |  | ■ | ■ | ■ | ■ | Total built-up area potentially exposed to floods in 1975 | EX_FD_B75 | km^2^ | (Florczyk et al. 2018) |  |  |
| **Disaster risk reduction** |  |  |  |  |  | Total built-up area potentially exposed to floods in 1990 | EX_FD_B90 | km^2^ |  |  |  |
|  |  |  |  |  |  | Total built-up area potentially exposed to floods in 2000 | EX_FD_B00 | km^2^ |  |  |  |
|  |  |  |  |  |  | Total built-up area potentially exposed to floods in 2015 | EX_FD_B15 | km^2^ |  |  |  |
|  |  |  |  |  |  | Total resident population potentially exposed to floods in 1975 | EX_FD_P75 |  |  |  | Number of people |
|  |  |  |  |  |  | Total resident population potentially exposed to floods in 1990 | EX_FD_P90 |  |  |  | Number of people |
|  |  |  |  |  |  | Total resident population potentially exposed to floods in 2000 | EX_FD_P00 |  |  |  | Number of people |
|  |  |  |  |  |  | Total resident population potentially exposed to floods in 2015 | EX_FD_P15 |  |  |  | Number of people |
|  | Storm surge exposure |  |  |  | ■ | Total surface potentially exposed to storm surges | EX_SS_AREA | km^2^ | JRC | Zonal statistics (sum) |  |
|  |  | ■ | ■ | ■ | ■ | Total built-up area potentially exposed to storm surges in 1975 | EX_SS_B75 | km^2^ | (Florczyk et al., 2018) | Zonal statistics (sum) |  |
|  |  |  |  |  |  | Total built-up area potentially exposed to storm surges in 1990 | EX_SS_B90 | km^2^ |  |  |  |
|  |  |  |  |  |  | Total built-up area potentially exposed to storm surges in 2000 | EX_SS_B00 | km^2^ |  |  |  |
|  |  |  |  |  |  | Total built-up area potentially exposed to storm surges in 2015 | EX_SS_B15 | km^2^ |  |  |  |
|  |  |  |  |  |  | Total resident population potentially exposed to storm surges in 1975 | EX_SS_P75 |  |  |  | Number of people |
|  |  |  |  |  |  | Total resident population potentially exposed to storm surges in 1990 | EX_SS_P90 |  |  |  | Number of people |
|  |  |  |  |  |  | Total resident population potentially exposed to storm surges in 2000 | EX_SS_P00 |  |  |  | Number of people |
|  |  |  |  |  |  | Total resident population potentially exposed to storm surges in 2015 | EX_SS_P15 |  |  |  | Number of people |
|  | Earthquake |  |  |  | ■ | Average peak ground acceleration (PGA) estimate of the seismic risk | EX_EQ19PGA | g | (Pagani et al. 2018) | Map algebra (max) | Acceleration (g) |
|  |  |  |  |  |  | MMI class of the seismic risk, derived from the PGA estimate | EX_EQ19MMI |  |  | Spatial join (feature) | Class (11) |
|  |  |  |  |  |  | Quality control value (available, missing – value not available, imprecise – not reliable estimate) | EX_EQ19_Q |  |  | Visual inspection | Class (12) |
|  | Heatwave | ■ 1980-2010 | | | | Maximum magnitude of the heatwaves | EX_HW_IDX |  | JRC | Map algebra | Index |

Supplementary Table 6 - Variables and attributes in the SDGs dimension

| **Dimension** | **Temporal coverage** | | | | **Variable** | **Attribute** | **GHS-UCDB** | **Metric** | **Input data** | **Method** | **Note** |
| --- | --- | --- | --- | --- | --- | --- | --- | --- | --- | --- | --- |
|  | **1975** | **1990** | **2000** | **2015** |  |  | **Field** |  |  |  |  |
| **Sustainable Development Goals** | ■ | | | | Land Use Efficiency (11.3.1) | Land use efficiency 1990-2015 | SDG_LUE9015 |  | (Melchiorri et al. 2019) | Join | Dimensionless |
|  | 1990-2015 | | | |  |  |  |  |  |  |  |
|  |  |  |  | ■ | Open spaces (11.7.1 –proxy) | Share of population living in the high green area in 2015 | SDG_A2G14 | share | JRC | Map algebra |  |
|  |  |  |  | ■ |  | Percentage of the open spaces | SDG_OS15MX | % | JRC | Map algebra |  |

**Notes:**

(1) Schema of Quality Code attribute: 0 – invalid; 1 – valid; 2 – uncertain.

(2) Major Geographical Regions (UN): Africa; Asia; Europe; Latin America and the Caribbean; Northern America; Oceania; Other – not classified

(3) Geographical Regions (UN): Australia/New Zealand; Caribbean; Central America; Central Asia; Eastern Africa; Eastern Asia; Eastern Europe; Melanesia; Micronesia; Middle Africa; Northern Africa; Northern America; Northern Europe; Polynesia; South America; South-Central Asia; South-Eastern Asia; Southern Africa; Southern Asia; Southern Europe; Western Africa; Western Asia; Western Europe; Other – not classified

(4) Name(s) source: GISCO and the full OpenStreetMap datasets; OTHER – web user feedback and other manual revisions.

(5) Biome type: Tropical and Subtropical Dry Broadleaf Forests; Mediterranean Forests, Woodlands, and Scrub; Temperate Grasslands, Savannas, and Shrublands; Deserts and Xeric Shrublands; Temperate Coniferous Forests; Tropical and Subtropical Coniferous Forests; Temperate Broadleaf and Mixed Forests; Boreal Forests/Taiga; Tropical and Subtropical Moist Broadleaf Forests; Tropical and subtropical grasslands, savannas, and shrublands; Flooded Grasslands and Savannas; Montane Grasslands and Shrubland; Mangroves; Tundra.

(6) Soil group (other): Acrisols; Alisols; Andosols; Anthrosols; Arenosols; Calcisols; Cambisols; Chernozems; Ferralsols; Fluvisols; Gleysols; Greyzems; Gypsisols; Histosols; Kastanozems; Leptosols; Leptosols; Lixisols; Luvisols; Nitisols; Phaeozems; Planosols; Plinthosols; Podzols; Podzoluvisols; Regosols; Solonchaks; Solonetz; Vertisols; Rock Outcrop; Sand Dunes; Water Bodies.

(7) Climate Classes: Desert (arid), and Cold arid; Desert (arid), and Hot arid; Mild temperate with dry summer, and Hot summer; Mild temperate with dry summer, and Warm summer; Mild temperate with dry winter, and Hot summer; Mild temperate with dry winter, and Warm summer; Mild temperate, fully humid, and Cool summer; Mild temperate, fully humid, and Hot summer; Mild temperate, fully humid, and Warm summer; Snow with dry summer, and Cool summer; Snow with dry summer, and Hot summer; Snow with dry summer, and Warm summer; Snow with dry winter, and Cool summer; Snow with dry winter, and Hot summer; Snow with dry winter, and Warm summer; Snow, fully humid, and Cool summer; Snow, fully humid, and Hot summer; Snow, fully humid, and Warm summer; Steppe (semi-arid), and Cold arid; Steppe (semi-arid), and Hot arid; Tropical monsoon; Tropical rain forest; Tropical savannah with dry summer; Tropical savannah with dry winter; Tundra.

(8) Major River Basins: Alabama River & Tombigbee; Amazonas; Amur; Apalachicola River; Aral Drainage; Armeria; Atrato; Balkhash; Bandama; Batang Hari; Batang Kuantan; Bei Jiang; Biobio; Brahmani River (Bhahmani); Brahmaputra; Brantas; Bravo; Brazos River; Buzi; Ca; Cape Fear River; Cauvery River; Cavally; Chao Phraya; Chelif; Chira; Chubut; Coco; Colorado (Argentinia); Colorado River (Caribbean Sea); Colorado River (Pacific Ocean); Columbia River; Comoe; Conception; Congo; Connecticut River; Cross; Cuanza; Cunene; Dalinghe; Damodar River; Danube; Daryacheh-Ye Orumieh; Daule & Vinces; Davo; Dead Sea; Delaware River; Dniepr; Dniestr; Don; Dong Jiang; Douro; Ebro; Elbe River; Escaut (Schelde); Esmeraldas; Fraser River; Fuchun Jiang; Fuerte; Galana; Gambia; Gamka; Ganges; Garonne; Geba; Gloma; Godavari; Grande De Matagalpa; Great Salt Lake; Grisalva; Groot- Kei; Groot-Vis; Guadalquivir; Guadiana; Han Jiang; Han-Gang (Han River); Hong(Red River); Huai He; Huang He (Yellow River); Hudson River; Incomati; Indus; Irrawaddy; Ishikari; Issyk-Kul; James River; Kelantan; Kiso; Kitakami; Kizilirmak; Kokemaenjoki; Kouilo; Krishna; Kuban; Kura; Kymijoki; Lake Chad; Lake Mar Chiquita; Lake Titicaca; Lake Turkana; Lake Vattern; Lempa; Lena; Liao He; Limari; Limpopo; Loa; Loire; Luan He; Lurio; Mae Klong; Magdalena; Mahanadi River (Mahahadi); Mahi River; Mamberamo; Maputo; Mekong; Merrimack River; Messalo; Min Jiang; Mira; Mississippi River; Mogami; Mono; Mucuri; Murray; Naktong; Narmada; Narva; Negro (Argentinia); Negro (Uruguay); Nelson River; Neman; Neva; Niger; Nile; Northern Dvina(Severnaya Dvina); Ntem; Nyong; Ob; Oder River; Ogooue; Okavango; Orange; Orinoco; Oueme; Oulujoki; Pangani; Panuco; Papaloapan; Paraiba Do Sul; Parana; Patacua; Pee Dee River; Penner River; Po; Potomac River; Pra; Pur; Purari; Pyasina; Rajang; Rapel; Rhine; Rhone; Rio Acara; Rio Capim; Rio De Contas; Rio Doce; Rio Gurupi; Rio Itapecur; Rio Jacui; Rio Jaguaribe; Rio Mearim; Rio Paraguac; Rio Paraiba; Rio Parnaiba; Rio Prado; Rio Ribeira Do Iguape; Roanoke River; Rogue River; Rovuma; Rufiji; Ruv; Sabine River; Sacramento River; Sakarya; Salado; Salinas; Salween; San Antonio River; San Joaquin River; San Juan; San Pedro; Sanaga; Santa; Santee River; Santiago; Sao Francisco; Sassandra; Savannah River; Save; Sebo; Seine; Senegal; Sepik; Shebelle; Shinano, Chikuma; Sittang River; Solo (Bengawan Solo); Southern Bug; St.Johns River; St.Lawrence; Sungai Kajan; Sungai Kapuas; Sungai Mahakam; Suriname; Susquehanna River; Tana; Tano; Tapti River; Tarim; Tejo; Tenry; Thames; Tigris & Euphrates; Tocantins; Tone; Tranh (Nr Thu Bon); Trent; Trinity River (Texas); Tsiribihina; Tugela; Tuloma; Ulua; Ural; Uruguay; Uwimb; Vaenern-Goeta; Van Gol; Verde; Volga; Volta; Vuoksi; Waikato River; Weser; Western Dvina (Daugava); Wisla; Xi Jiang; Yangtze River (Chang Jiang); Yaqui; Yenisei; Yodo; Yongding He; Zambezi.

(9) Income classes: HIC - High Income Countries; UMIC - Upper-middle Income Countries; LMIC - Lower-middle Income Countries; LIC - Low Income Countries.

(10) Development Groups: MDR - More Developed Regions; LCD - Less developed regions, excluding least developed countries; LDCL - Least developed countries.

(11) MMI classes: from 1 to 8.

(12) Quality control of the earthquake data: available; missing – value not available; imprecise – not reliable estimate

**REFERENCES**

Corbane, C., Pesaresi Martino, Politis Panagiotis, Florczyk J. Aneta, Melchiorri Michele, Freire Sergio, Schiavina Marcello, Ehrlich Daniele, Naumann Gustavo, and Kemper Thomas. 2018. “The Grey-Green Divide: Multi-Temporal Analysis of Greenness across 10,000 Urban Centres Derived from the Global Human Settlement Layer (GHSL).” *International Journal of Digital Earth*, October, 1–18. https://doi.org/10.1080/17538947.2018.1530311.

Crippa, Monica, Diego Guizzardi, Marilena Muntean, Edwin Schaaf, Frank Dentener, John A. van Aardenne, Suvi Monni, et al. 2018. “Gridded Emissions of Air Pollutants for the Period 1970–2012 within EDGAR v4.3.2.” *Earth System Science Data* 10 (4): 1987–2013. https://doi.org/10.5194/essd-10-1987-2018.

Dottori, Francesco, Peter Salamon, Alessandra Bianchi, Lorenzo Alfieri, Feyera Aga Hirpa, and Luc Feyen. 2016. “Development and Evaluation of a Framework for Global Flood Hazard Mapping.” *Advances in Water Resources* 94 (August): 87–102. https://doi.org/10.1016/j.advwatres.2016.05.002.

Fischer, G., F. Nachtergaele, S. Prieler, H. T. van Velthuizen, L. Verelst, and D. Wiberg. 2008. “Global Agro-Ecological Zones Assessment for Agriculture (GAEZ 2008).” IIASA, Laxenburg, Austria and FAO, Rome, Italy.

Florczyk, A., D. Ehrlich, C. Corbane, S. Freire, T. Kemper, M. Melchiorri, M. Pesaresi, P. Politis, M. Schiavina, and L. Zanchetta. 2018. “Community Pre-Release of GHS Data Package (GHS CR2018) in Support to the GEO Human Planet Initiative.” *Publications Office of the European Union*. https://doi.org/10.2760/7778.

Global Burden of Disease Collaborative Network. 2018. “Global Burden of Disease Study 2017.” Institute for Health Metrics and Evaluation (IHME). http://ghdx.healthdata.org/record/ihme-data/gbd-2017-population-estimates-1950-2017.

Global Runoff Data Centre. 2007. “Major River Basins of the World.” https://www.bafg.de/GRDC/EN/02_srvcs/22_gslrs/221_MRB/riverbasins.html?nn=201570.

Harris, I., P.D. Jones, T.J. Osborn, and D.H. Lister. 2014. “Updated High-Resolution Grids of Monthly Climatic Observations - the CRU TS3.10 Dataset: UPDATED HIGH-RESOLUTION GRIDS OF MONTHLY CLIMATIC OBSERVATIONS.” *International Journal of Climatology* 34 (3): 623–42. https://doi.org/10.1002/joc.3711.

Melchiorri, Michele, Martino Pesaresi, Aneta J. Florczyk, Christina Corbane, and Thomas Kemper. 2019. “Principles and Applications of the Global Human Settlement Layer as Baseline for the Land Use Efficiency Indicator—SDG 11.3.1.” *ISPRS International Journal of Geo-Information* 8 (2): 96. https://doi.org/10.3390/ijgi8020096.

OECD Environment Statistics (database). 2018. “Air Quality and Health: Exposure to PM2.5 Fine Particles - Countries and Regions.” OECD. https://doi.org/10.1787/96171c76-en.

Olson, David M., Eric Dinerstein, Eric D. Wikramanayake, Neil D. Burgess, George V. N. Powell, Emma C. Underwood, Jennifer A. D’amico, et al. 2001. “Terrestrial Ecoregions of the World: A New Map of Life on Earth.” *BioScience* 51 (11): 933. https://doi.org/10.1641/0006-3568(2001)051[0933:TEOTWA]2.0.CO;2.

Pagani, Marco, Julio García-Pelaez, Robin Gee, Kendra Johnson, Valerio Poggi, Michele Simionato, Richard Styron, et al. 2018. “GEM Global Seismic Hazard Map v.2018.1.” https://doi.org/10.13117/GEM-GLOBAL-SEISMIC-HAZARD-MAP-2018.1.

Rubel, Franz, Katharina Brugger, Klaus Haslinger, and Ingeborg Auer. 2017. “The Climate of the European Alps: Shift of Very High Resolution Köppen-Geiger Climate Zones 1800–2100.” *Meteorologische Zeitschrift* 26 (2): 115–25. https://doi.org/10.1127/metz/2016/0816.

Tadono, T., H. Nagai, H. Ishida, F. Oda, S. Naito, K. Minakawa, and H. Iwamoto. 2016. “Generation of the 30 M-Mesh Global Digital Surface Model by Alos Prism.” *ISPRS - International Archives of the Photogrammetry, Remote Sensing and Spatial Information Sciences*, June, 157–62. https://doi.org/10.5194/isprs-archives-XLI-B4-157-2016.

Weiss, D. J., A. Nelson, H. S. Gibson, W. Temperley, S. Peedell, A. Lieber, M. Hancher, et al. 2018. “A Global Map of Travel Time to Cities to Assess Inequalities in Accessibility in 2015.” *Nature* 553 (7688): 333–36. https://doi.org/10.1038/nature25181.
